# A protein-coated micro-sucker patch inspired by octopus for adhesion in wet conditions

**DOI:** 10.1038/s41598-020-72493-7

**Published:** 2020-09-23

**Authors:** Gabriella Meloni, Omar Tricinci, Andrea Degl’Innocenti, Barbara Mazzolai

**Affiliations:** 1 Center for Micro-BioRobotics, Istituto Italiano di Tecnologia, Viale Rinaldo Piaggio 34, 56025 Pontedera (Pisa), Italy; 2 The BioRobotics Institute, Scuola Superiore Sant’Anna, Viale Rinaldo Piaggio 34, 56025 Pontedera (Pisa), Italy; 3 Smart Bio-Interfaces, Istituto Italiano di Tecnologia, Viale Rinaldo Piaggio 34, 56025 Pontedera (Pisa), Italy

**Keywords:** Biomedical engineering, Marine biology

## Abstract

In medical robotics, micromanipulation becomes particularly challenging in the presence of blood and secretions. Nature offers many examples of adhesion strategies, which can be divided into two macro-categories: morphological adjustments and chemical adaptations. This paper analyzes how two successful specializations from different marine animals can converge into a single biomedical device usable in moist environments. Taking inspiration from the morphology of the octopus sucker and the chemistry of mussel secretions, we developed a protein-coated octopus-inspired micro-sucker device that retains in moist conditions about half of the adhesion it shows in dry environments. From a robotic perspective, this study emphasizes the advantages of taking inspiration from specialized natural solutions to optimize standard robotic designs.

## Introduction

In robotics, a fundamental aspect of grippers and micromanipulators is adhesion; improving adhesion capabilities of robotic devices can simplify the manipulation of compliant or slippery objects^1–4^. Adhesion is also a basic requirement for the locomotion of climbing robots^5–7^. In the last years, taking inspiration from adhesive structures diffused in nature to develop artificial adhesion solutions became a common practice. Several organisms are naturally provided with different strategies for adhesion, depending on their habitat and on the physiological function of the attachment^8,9^. *Galium aparine*, for instance, is a climbing plant that anchors to substrates mechanically, by leveraging its hooks^10^; geckos exploit fibrillar matrices covering their pads to adhere to vertical or inverted surfaces^11–13^. A high number of adhesive robots have been derived from biological models, suggesting a general validity for the method^14–20^.

When looking at different natural strategies to implement adhesive artifacts, it is important to consider the characteristics of the environment in which one wants to operate. An organ evolved for dry adhesion might be not as successful in wet conditions, and vice versa. For biomedical applications, the challenge is often to develop new materials and devices that solidly interact with different tissues without causing damage.

In nature, adhesion strategies can be divided into two macro-categories: those depending on morphology and others based on chemical interactions. Among the natural solutions for wet adhesion relying on morphology, a remarkable example is the octopus sucker (here we refer in particular to *Octopus* spp*.*). This is a flexible organ deputed to a reversible adhesion to different materials, with a crucial role for sensing, grasping and body anchoring^21–26^. A sucker is composed by two distinct structures: an external cup with an exposed disk-like portion, named *infundibulum*, connected to an *acetabulum*, a more internal chamber with a central protuberance^22,23,27^. To reach vacuum, at first infundibular muscles are contracted, maximizing the contact area between infundibulum and substrate. Then, acetabular muscles contract, both to push out water inside the cup and to minimize space between the infundibulum and the acetabular protuberance, generating increased friction and a negative force^27,28^. The key aspect for the adhesion of such a structure is conformability, which depends on peculiar morphology and mechanical properties that allow the sucker to adapt to various objects and textures. Throughout the octopus skin, there is a poorly characterized mucus possibly composed of mucopolysaccharides and glycoproteins, which may facilitate adhesion^23,29^. Instances of artificial octopus-inspired suckers are already available, each one implementing different aspects of their biological counterpart. Some of these devices focus on the morphology of a single sucker, the presence of grooves in the infundibulum, and the role of vacuum in the adhesion^21,30^. The actuator developed by Follador et al.^31^ exploits the passive deformation of a sucker for the activation of the device. Suckers are also present in a gripper proposed by Tomokazu et al.^32,33^, in which they ensure an excellent grasp of objects with different shapes. All these examples need an external vacuum pump either to activate the grabbing^32^ or just to maintain a stable internal pressure^21,30,34^. Other devices only mimic the compliant design of an octopus sucker, as in the case of adhesive patches that rely solely on the deformation of miniaturized suckers when external pressure is applied^16,35,36^. Chun et al.^37^ use this strategy to develop a sensorized wearable electronic gear for health monitoring. The aforementioned examples of octopus-mimicking designs confirm an increasing interest in octopus bioinspiration for new solutions in robotics and medical care.

Another mollusk, the mussel *Mytilus edulis*, utilizes a different mechanism to chemically adhere to submerged or moist substrates: the secretion of adhesive proteins. In mussels, adhesion is mediated by *byssus*, a coriaceous bundle of protein filaments that builds a plaque anchoring the mussel to various surfaces^38–40^. The adhesive proteins involved in byssus formation are known as mussel foot proteins (mfps). The gluing power of these proteins is due to the presence of the amino acid 3,4-dihydroxy-l-phenylalanine, an unusual form of hydroxylated tyrosine^41^. There are different types of mfps; their capacity to adhere depends on the proportion of 3,4-dihydroxy-l-phenylalanine residues, with the mussel foot protein 1 (mfp-1) being the stickiest mfp known to date^38,42^. Byssus already influenced a few robotic applications, such as an adhesive inspired to the pads of geckos and coated with a molecule that imitates the sticky proteins of mussels^39^. The mussel foot attachment has also been studied to develop new materials that aid the self-healing of wounds^38,43^.

With this work, we present a device that takes inspiration from both the morphology of the octopus sucker and the chemical properties of the mussel foot, for a reversible adhesion in wet conditions. Our prototype is composed of an array of micro-suckers coated with an mfp; it is soft and highly biocompatible, allowing for a safe interaction with biological tissues. In comparison with previous wet-tolerant patches^16,35^, our device may prove especially useful when a high level of reproducibility is desired, both between single micro-suckers in a given array and in terms of standardization of different fabrications.

Among other potential uses, wet-tolerant devices like the one we propose are helpful supplements for common medical robotic platforms, to provide reliable attachment to tissues when blood and secretions are present. Adhesive patches also promote the regeneration of wounds, and may become part of body sensors or drug delivery systems even in particularly moist locations, such as the eye^36,37,44,45^.

## Results

### Device fabrication

**Figure 1:**
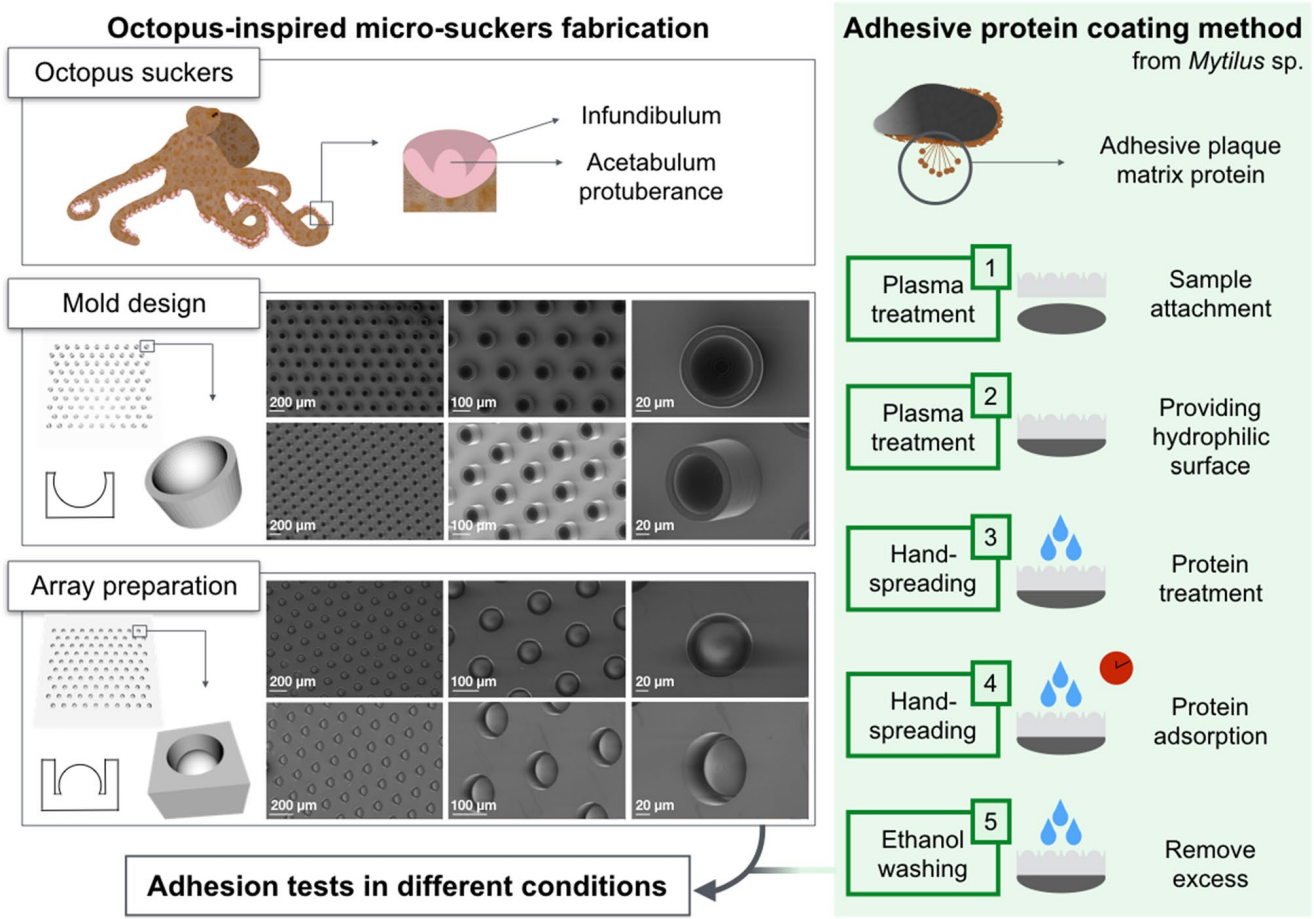
General framework. Octopus arms with suckers; a magnified box depicts a sucker section. The “Mold design” box shows the mold array model, with a three-dimensional model of a single mold/sucker (magnified box) and the simplified profile of a single cavity (on the left). SEM images portray a mold array in orthogonal (top lane) or oblique (bottom lane) view. The “Array preparation” box shows the final array model, with a three-dimensional model of a single sucker-like structure (magnified box) and the simplified profile of a single cavity (on the left). SEM images retract a PDMS array in orthogonal (top lane) or oblique (bottom lane) view. The green box shows the outline of the coating method with the adhesive protein mfp-1.

Millimetric square arrays of micro-suckers in polydimethylsiloxane (PDMS) were obtained from molds produced by means of direct laser lithography; each sucker is composed of a pillar harboring a spherical infundibulum-like bulge, as shown in Fig. 1. Flat PDMS surfaces served as negative controls for all downstream procedures.

### Preliminary optimizations of protein coating

**Figure 2:**
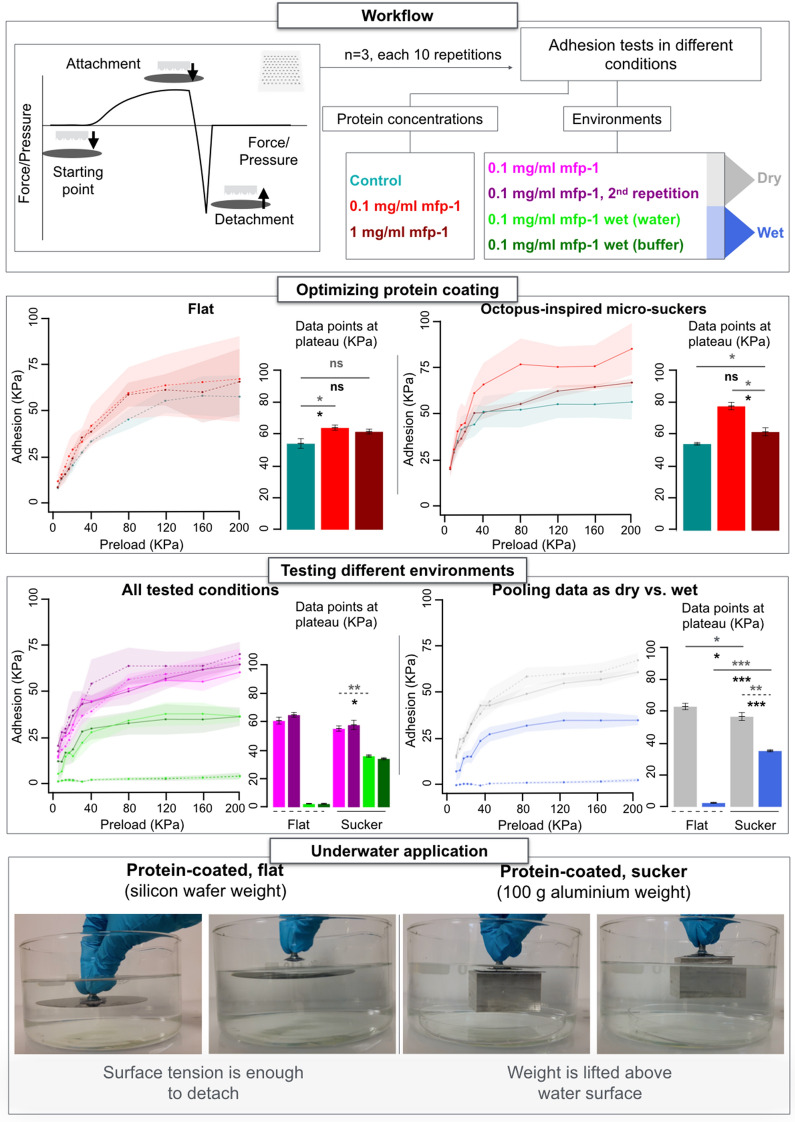
Adhesion tests in different conditions. The top-left box contains a simplistic force vs. time diagram, in which the detachment moment corresponds to the adhesion force; all tested conditions are listed, each one represented with different colors. Each condition was tested using three different samples (n = 3), and every sample was tested for ten repetitions. For line charts, dashed curves are used for control experimental classes with flat surfaces. Solid curves mark experimental classes with octopus-inspired micro-suckers. Data dispersions, visualized as ribbons, report standard error of the mean. Bar charts feature averages of the four data points at the right-end (i.e. at plateau) of every curve. The number of asterisks specifies the significance level for different p-value thresholds (ns—i.e. non-significant—for p > 0.05, *for p < 0.05, **for p < 0.01, ***for p < 0.001); solid lines indicate unpaired two-tailed t-test, and dashed lines paired two-tailed t-test. Line ends indicate the two bars compared in each test. Gray asterisks are used for t-tests, and black asterisks for two-tailed Mann–Whitney U-tests; error bars report standard error of the mean. The bottom box represents the underwater application. Images are screenshots from the demonstrative Supplementary Video S1.

Some samples were coated with an mfp-1 solution; others received instead a control treatment. We initially tested two different protein concentrations (1 mg/ml and 0.1 mg/ml mfp-1 coating solution), and carried out adhesion tests (n = 3, ten repetitions each, Fig. 2) in a dry environment, quantifying attachment as a function of imposed preload pressures.

In these conditions, adhesion is mildly enhanced in the presence of proteins, and this becomes more evident for micro-sucker arrays vs. control (flat) surfaces (Fig. 2). Best results were achieved with the 0.1 mg/ml mfp-1 coating solution. In turn, micro-suckers per se do not seem to have major effects under tested circumstances.

### Adhesion tests in dry and wet environments

Further adhesion experiments (n = 3, ten repetitions each, Fig. 2) were invariably performed in the presence of protein coating (coating solution 0.1 mg/ml mfp-1), again for micro-sucker arrays or flat PDMS surfaces as controls. Each sample was first tested in dry conditions; in order to probe protein coating stability, a second dry experiment was then performed. Two other tests served to assess adhesion properties when a drop of fluid was added: the first was carried with deionized water, the second one with a saline buffer at pH 7.5, closer to the natural marine environment^46^.

Adhesion does not visibly decrease when an experiment in dry conditions is repeated. The two different wet experiments yield comparable results. Suckers do not seemingly offer particular advantages in dry circumstances under experimental settings. However, when some moisture is added the system clearly needs suckers to retain relevant adhesive properties. This becomes particularly obvious regrouping data into two macro-categories, namely all dry and all wet experiments (Fig. 2).

### Demonstrative underwater application

As a practical demonstration, we tested a wider array of protein-coated micro-suckers underwater. The array was attached to a grip, then pushed by hand against a submerged weight found in the bottom of a beaker, and slowly lifted vertically beyond the water surface; after that, the detachment was attempted by peeling off the array.

Our device was able to collect a silicon wafer, as well as a 100 g aluminum block; tilting the grip proved sufficient to achieve intentional detachment. An analogous flat device, tested in identical conditions, failed already to collect a silicon wafer, see Fig. 2 and Supplementary Video S1.

## Discussion

The present work highlights the possibility to implement protein-coated octopus-inspired micro-suckers in robots and plasters to improve object adhesion in moist environments. Our device achieves underwater attachment by combining morphological adaptations of octopuses with molecular features of mussels.

Consistent with literature, we found that the protein alone displays relevant adhesive properties. Micro-suckers do not offer particular advantages in the tested dry conditions. However, at least in wet environments the protein coating becomes more effective when micro-suckers are present; one might think this is because suckers increase surface, but the most effective protein concentration was the lowest investigated, rather suggesting a synergy between the physical adhesion provided by suckers and the chemical stickiness attributable to mfp-1. Confirming the idea that impurities might alter the functioning of a morphology-based adhesive, the decreased performance of the higher tested concentration vs. the lower one hints to an excess of protein clogging suckers. An ideal protein concentration should trade off gluing effects against disturbance of the mechanical action of suckers. Fine tuning might enhance performances, and will also depend on final use. Adhesion stands multiple experiments, showing at least some robustness of the design. Unexpectedly, the use of a saline buffer instead of pure water was unnecessary under our settings.

In a moist environment, protein plus micro-suckers are ~ 14 times more adhesive than a flat surface with protein; roughly fifty percent of the dry adhesion is retained when protein-coated micro-suckers are exposed to moisture. The adhesion of flat devices in water drops instead to almost nothing also in the presence of mfp-1, and this is evident in practical demonstrations too: a flat sample could not retain grip even when presented with minor disturbances, i.e. the reaching of the water surface.

Likely due to positional effects when placing samples, the use of a single sample for different experimental classes reduce data dispersion; this indicates that the direction of pressure is important, namely that pushing must be orthogonal to the surface. While this property might be a limitation for some applications, it might turn useful for others, like reversible adhesion. In fact, at the end of our demonstrative video we intentionally detached the recovered objects.

Silicone materials have been widely used in medical applications. In particular, PDMS satisfies standard criteria for biocompatibility not causing irritation and sensitization when in contact with biological tissues. In fact, PDMS-based devices have also been approved for long-term usage implants, also thanks to the introduction of anti-bacterial treatments^47–49^.

The proven biosafety of PDMS enables the deployment of our proposed protein-coated micro-sucker adhesive patch in different medical practices, such as long-term implants for drug delivery, wound regeneration and body sensors, or in robotic platforms for surgical procedures, particularly in moist or wet conditions rather than in dry environments.

## Methods

### Fabrication

The design of a single octopus-inspired micro-sucker was modeled with Blender (Blender Foundation). It consists of a cylindrical cavity with a depth of 75 μm and a diameter of 100 μm. Inside this cavity, a spherical bulge of 85 μm in diameter and 65 μm in height is found. The design of the mold was obtained from the negative of the model of the sucker. Molds were fabricated in IP-S photoresist (Nanoscribe GmbH) on glass substrates, by means of direct laser lithography (Photonic Professional system, Nanoscribe GmbH). For every mold, the glass substrate was rinsed with isopropyl alcohol and deionized water, and the negative tone IP-S photoresist was cast on it. The writing configuration of the Photonic Professional system was with the objective (25 ×, NA 0.8) in immersion in the photoresist. The mold was fabricated by exposing the photoresist to a laser beam (Calman laser source) at a center wavelength of 780 nm, using a writing speed of 15 mm/s with a power of 68.4 mW. The sample was developed for 20 min in SU-8 Developer (MicroChem Corp) and rinsed in isopropyl alcohol and deionized water. The final result was a square array mold of microstructures following a hexagonal lattice pattern with a spacing of 200 μm between the centers of each sucker. The area measured 25 mm^2^, except for those patches used for practical demonstrations, which had an area equal to 1 cm^2^.

Each PDMS patch was produced by means of a micro-molding technique from the micro-fabricated mold. The mold was chemically functionalized by means of silanization in order to ensure an easy detachment of the PDMS: the surface was activated in air plasma for 1 min at 50 W, and evacuated to 650 mbar below atmosphere together with 3 ml solution 0.3% v/v of Trichloro(1H,1H,2H,2H-perfluorooctyl)silane in cyclohexane. PDMS (monomer and reticulation agent in a 1:10 ratio) was cast onto the mold in a Petri dish, reaching a thickness of 1 mm. The curing was carried out for 24 h at room temperature in vacuum. The sample for the adhesion tests was cut with a surgical blade under an optical microscope (Fig. 1). A 25 mm^2^ PDMS array was totally composed of 168 micro-suckers. To verify the standardization of the fabrication process, micrographs of both molds and casts were taken with a scanning electron microscope (SEM).

For each sample, a flat control was cut from the same cast in a region without micro-suckers, in order to reduce fabrication variability between adhesive samples and relative controls. Each mold was used several times after rinsing and subsequent silanization.

To achieve a permanent and stable attachment of the PDMS patch to its support (a silicon wafer), a plasma treatment was performed on both ends. Plasma exposes hydroxyl groups on the silicon wafer and silanol groups on PDMS, allowing the formation of a strong Si–O–Si covalent bond. As PDMS is a hydrophobic material, a second plasma treatment was performed to provide a hydrophilic surface. This method, known as *hydrophilic functionalization*, also increases biomolecular adsorption^50^. Both treatments were carried out in an oxygen plasma system for 30 s, at a power of 30 W and a pressure of 20 mbar.

### Protein coating

PDMS patches were coated with *Mytilus edulis* mussel foot protein 1, mfp-1, commercially available as Native Mussel Adhesive protein (ab155708, Abcam); the stock concentration was 1 mg/ml in 1% v/v of acetic acid in deionized water. Two concentrations of protein were chosen, namely 1 and 0.1 mg/ml, invariably with 1% acetic acid. A plain 1% acetic acid solution was used as a control. A low amount of liquid (20 µl for 25 mm^2^ and 80 µl for 1 cm^2^) was spread on the patch surface. Solutions were kept for 10 min under a chemical hood, enabling the evaporation of the acetic acid. Afterward, surfaces were washed with absolute ethanol to remove excess protein (Fig. 1).

### Adhesion tests

Adhesion experiments were conducted with a custom-built multi-axis measurement platform integrated with a loading cell (ATI, Nano17), at room temperature. Each sample was mounted by its support on a metal screw, and then orthogonally pushed against a silicon wafer with different preloads (4, 8, 12, 16, 20, 30, 40, 80, 120, 160, or 200 kPa). Whenever the imposed preload pressure was reached the loading cell started detachment. Within a single experiment, every preload pressure was tested ten times (Fig. 2).

We first performed a set of experiments in dry conditions, testing both protein concentrations. More precisely, we assessed adhesion for micro-sucker arrays and their flat controls in presence or absence of protein coating, in the latter case either at 1 or 0.1 mg/ml starting concentration. Different PDMS patches were used for each of three repetitions.

A second set of tests was conducted exclusively on protein-coated (0.1 mg/ml starting concentration) samples, namely micro-suckers arrays and their flat controls. For each of three repetitions, this time a single PDMS patch was used for all different experimental classes: at first, an experiment in dry conditions was performed twice; a third test was conducted after putting a 100 µl drop of deionized water on the area of the silicon wafer entering in contact with the sample. Finally, we added 100 µl of a saline buffer (0.1 M acetate, 0.6 M NaCl in PBS with a pH 7.5) where we previously placed the water, and a further experiment was carried out.

### Data analysis

Plots were obtained via local scripting. We produced line charts showing mean attachment pressures (± standard error of the mean) as a function of imposed preloaded pressures. Averaging the four highest mean attachment pressures for each curve, we also produced bar charts, reporting data dispersion as standard error of the mean. Two-tailed independent and dependent t-tests and Mann–Whitney U-test were performed when needed.

### Demonstrative video

A crystallizer was filled with deionized water to demonstrate the ability of our patch to collect objects in water. A protein-coated flat device and a protein-coated micro-sucker device (each 1 cm^2^ broad, with 0.1 mg/ml mfp-1 starting concentration) were mounted on a metal screw as a support holder. Both devices were tested by hand for the collection of a silicon wafer (7.62 cm in diameter, 380 µm in thickness) and a rectangular aluminum weight of 100 g (30 × 30 × 41 mm). In case the weight was successfully lifted beyond the water surface, the detachment was attempted by tilting the support to one side. The procedure was video-recorded for demonstration.

## Supplementary information

Supplementary Legends

Supplementary Video S1.

## Data Availability

All relevant data are included within the article and its supplementary information.
