# Supplementary material for: A protein-coated micro-sucker patch inspired by octopus for adhesion in wet conditions
Source: Sci Rep. 2020 Sep 23;10:15480. doi: 10.1038/s41598-020-72493-7 (PMC7511962; doi:10.1038/s41598-020-72493-7)
Supplement: Supplementary file 1 — Supplementary Legends [file 41598_2020_72493_MOESM1_ESM.docx]

**Supplementary information**

**Supplementary Video S1. Demonstrative underwater application.** Example of a protein-coated micro-sucker device (1 cm^2^ broad, with 0.1 mg/ml mfp-1) lifting a silicon wafer and a rectangular aluminum weight of 100 g beyond the water surface, in comparison to a flat protein-coated surface that already fails at retrieving a silicon wafer from water. For the micro-sucker device, the load could be detached simply by peeling off the patch.
